# The Role of Epigenetic Change in Autism Spectrum Disorders

**DOI:** 10.3389/fneur.2015.00107

**Published:** 2015-05-26

**Authors:** Yuk Jing Loke, Anthony John Hannan, Jeffrey Mark Craig

**Affiliations:** ^1^Murdoch Childrens Research Institute, Royal Children’s Hospital and Department of Paediatrics, University of Melbourne, Parkville, VIC, Australia; ^2^Melbourne Brain Centre, Florey Institute of Neuroscience and Mental Health, The University of Melbourne, Parkville, VIC, Australia

**Keywords:** epigenetics, methylation, autism spectrum disorders, epigenomics, gene expression

## Abstract

Autism spectrum disorders (ASD) are a heterogeneous group of neurodevelopmental disorders characterized by problems with social communication, social interaction, and repetitive or restricted behaviors. ASD are comorbid with other disorders including attention deficit hyperactivity disorder, epilepsy, Rett syndrome, and Fragile X syndrome. Neither the genetic nor the environmental components have been characterized well enough to aid diagnosis or treatment of non-syndromic ASD. However, genome-wide association studies have amassed evidence suggesting involvement of hundreds of genes and a variety of associated genetic pathways. Recently, investigators have turned to epigenetics, a prime mediator of environmental effects on genomes and phenotype, to characterize changes in ASD that constitute a molecular level on top of DNA sequence. Though in their infancy, such studies have the potential to increase our understanding of the etiology of ASD and may assist in the development of biomarkers for its prediction, diagnosis, prognosis, and eventually in its prevention and intervention. This review focuses on the first few epigenome-wide association studies of ASD and discusses future directions.

## Introduction

Autism spectrum disorders (ASD) are defined diagnostically by impaired social communication, restricted interests, and repetitive behaviors, defined hereafter as endophenotypes. Such endophenotypes are thought to result from disordered neurodevelopment, although the precise etiology is unknown (1–3). Other common, but not universal, features of ASD include attention deficits, sensory and motor abnormalities, cognitive impairment, and epilepsy (1, 3). Gastrointestinal dysbiosis and impaired immune and mitochondrial function have been observed in subsets of individuals with ASD, although the association of these endophenotypes with neurodevelopment is yet unclear (see also Section “The Genetics of Autism”). ASD have a prevalence of approximately 1% and strong male bias (male:female ratio approximately 4:1).

The reference standard tools for diagnosing ASD using a multidisciplinary team include the Diagnostic and Statistical Manual of Mental Disorders (DSM) and the International Statistical Classification of Diseases and Related Health Problems (4, 5). To aid diagnosis, other tools have been developed, with the most widely used being the Autism Diagnostic Interview-Revised (ADI-R) and the Autism Diagnostic Observation Schedule (ADOS) (reviewed in Ref. (6)). Another tool sometimes used as an initial screen in childhood, the Childhood Autism Spectrum Test (CAST), is a questionnaire that measures social and communication skills in a non-clinical setting (7).

## The Genetics of Autism

**Figure 1:**
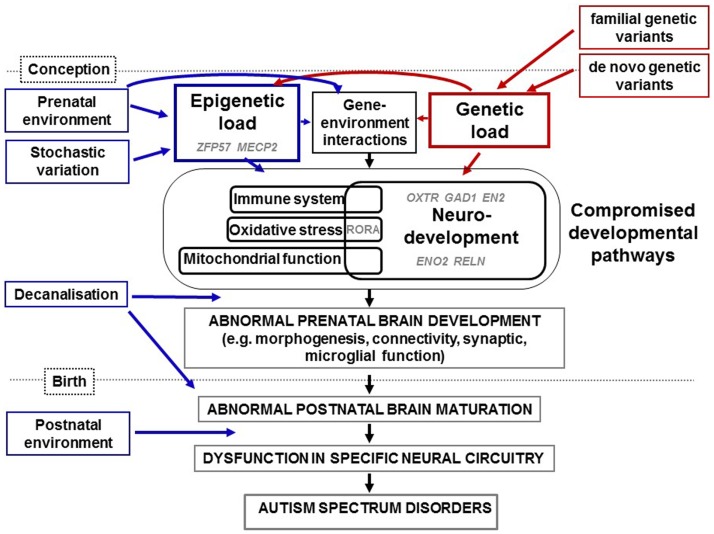
**Diagrammatic representation of how genetic and epigenetic changes combine and interact in the etiology of ASD as summarized in the text**. Epigenetic load (from prenatal environment and stochastic variation) and genetic load (from familial and *de novo* variation) interact to compromise neurodevelopmental, immune, oxidative stress, and mitochondrial pathways identified through studies of ASD genetics, physiology, expression, and/or methylation. We have highlighted in gray the putative involvement of specific genes mentioned in this review for which evidence has come from candidate and genome-wide studies. Examples of genes implicated in epigenetic load for ASD are *ZFP57* and *MECP2*. In addition, sequence variants of genes involved in the control of expression such as *ADNP*, *ASH1L*, *CHD8*, and *ARID1B* (not shown), will induce epigenetic changes within genes that they regulate. Evidence has come from studies of epigenetics and gene expression for the dysregulation of *OXTR*, *GAD1*, *RELN*, *EN2*, and *ENO2* during neurodevelopment in ASD. Further interactions will occur between specific genes and specific prenatal environments. Over a certain threshold of genetic and epigenetic dysfunction, development is decanalized and neurodevelopment disrupted. This includes defects in synaptic function, connectivity, and morphogenesis and would lead to abnormal brain maturation, neural circuity dysfunction, characteristic endophenotypes of ASD. Further, postnatal environments may also contribute to severity of symptoms.

Autism spectrum disorders, like all other human conditions and diseases, are likely caused by a combination of genes, environment, and the interaction between the two (Figure 1). The degree of variation in ASD phenotype caused by genetic variation has been estimated at between 40 and 90% ((8) and references therein). Specific genetic variations in ASD have been studied by measuring single nucleotide variants (SNVs), copy number variants (CNVs) and cytogenetic abnormalities (reviewed in Refs. (9–11)). To date, hundreds of genes have been linked to ASD using such approaches. However, reproducible genetic variants have been linked with a very small percentage of cases of non-syndromal ASD. Although this underscores the phenotypic heterogeneity of ASD, we now know that common variants of small effect and rare and *de novo* variants of large effect can combine to influence risk for ASD (12). For an up-to-date database of ASD-associated genes, the reader is referred to the Simons Foundation Autism Research Initiative (SFARI) ASD gene database (https://gene.sfari.org/ (13)). This resource reviews the evidence for each gene’s association with ASD and assigns a score, from 6 (“not supported”) to 1 (“high confidence”). Genes associated with syndromic ASD are categorized separately. As of April 2015, there were 667 genes in this database, with 16 listed as “high confidence” (Table 1). Some, such as *CHD8*, *DYRK1A*, and *TBR1* are active in early development. Of particular interest, half of these genes have a proven or suspected function in networks that involve epigenetic change, such as transcription factors (e.g., *ADNP*, *ASH1L*, *CHD8*) and ATP-dependent chromatin remodellers (e.g., *ARID1B*). This reflects the findings of many genome-wide genetic studies (14–18). A second major genetic network identified in ASD is neurodevelopment, including cell signaling (15, 16, 18). As early development is accompanied by rapid epigenetic change, many genes are likely to function in both pathways. This is evidenced by dual functions for genes such as *CHD8* (19) and *ARID1B* ((20) and discussed in Ref. (21)). A third frequently identified ASD-associated genetic network involves synaptic function, including related networks of postsynaptic density protein complexes, neuronal cell adhesion and the balance of excitation and inhibition (9, 10, 16–18, 22). This is likely to be due at least in part to genes with a dual role in synaptic development and function (reviewed by Chen et al. (23)). One other network highlighted by genetic studies of ASD is mitochondrial function (16) and evidence suggests that disruption of this network may compromise neurodevelopment, agreeing with the elevated levels of mitochondrial disease seen in ASD (reviewed in Ref. (24)). It is also important to note that, via protein–protein interactions and the regulation of multiple genes by sequence-specific factors, all of the above networks are in some way interconnected (25). In Figure 1, we summarize these genetic pathways to ASD via disrupted neurodevelopmental and other gene pathways.

**Table 1:** **Genes for which there is high confidence of association with ASD from genetic evidence (https://gene.sfari.org/ (13))**.

Abbreviation	Name	Protein function
ADNP	Activity-dependent neuroprotector homeobox	Vasoactive intestinal peptide, neuroprotective factor, transcription factor (E)
ANK2	Ankyrin 2, neuronal	Cytoskeletal and cell membrane protein
ARID1B	AT rich interactive domain 1B (SWI1-like)	ATP-dependent chromatin remodeller (E)
ASH1L	Ash1 (absent, small, or homeotic)-like (Drosophila)	Transcriptional activator, cell-cell tight junctions (E)
ASXL3	Additional sex combs like 3 (Drosophila)	Possible regulator of transcription (E)
CHD8	Chromodomain helicase DNA binding protein 8	Transcriptional repressor involved in early development (E)
DYRK1A	Dual-specificity tyrosine-(Y)-phosphorylation regulated kinase 1A	Protein kinase involved in signaling and early development
GRIN2B	Glutamate receptor, inotropic, *N*-methyl d-aspartate 2B	Glutamate receptor involved in long-term potentiation and synaptic transmission
POGZ	Pogo transposable element with ZNF domain	Possible transposase and transcription factor (E)
PTEN	Phosphatase and tensin homolog (mutated in multiple advanced cancers 1)	Tumor suppressor involved in signaling and mitochondrial function
SCN2A	Sodium channel, voltage-gated, type II, alpha subunit	Sodium channel expressed in the brain
SETD5	SET domain containing 5	Likely chromatin protein (E)
SHANK3	SH3 and multiple ankyrin repeat domains 3	Postsynaptic density synapse scaffold protein
SUV420H1	Suppressor of variegation 4-20 homolog 1 (Drosophila)	Likely chromatin protein (E)
SYNGAP1	Synaptic Ras GTPase activating protein 1	Postsynaptic density synapse protein
TBR1	T-box, brain, 1	Likely transcription factor associated with early cortical development (E)

*(E) indicates a proven or likely epigenetic function based on published data (reviewed in Ref. (14–18))*.

It has been proposed that, rather than resulting from dysfunction of specific genes, ASD result from the dysfunction of specific genetic pathways (26), an extension of an early “polygenic hypothesis” for the origins of neurodevelopmental disorders (27). These ideas also agree with the threshold model of disease (28, 29) in which ASD phenotypes become apparent only after a certain burden of genetic risk alleles has been reached; burden could apply within specific pathways too. These ideas could help explain the apparent genetic heterogeneity of ASD and may lead to potential therapies based on these pathways. The story may be yet more intricate; complex eukaryotes have inbuilt genetic redundancy and homeostatic mechanisms and can buffer or compensate for the loss of a particular gene within a pathway (30, 31). However, such homeostatic mechanisms may not always fully compensate, resulting in subtle and possibly tissue-specific maladaptive phenotypic change, defined as decanalization (32). This concept is based on Waddington’s original definition of canalization as the ability of an organism to maintain phenotypic fidelity in the face of environmental and/or genetic perturbation (33). It has been suggested that the recently evolved neocortex may not have had time to develop such robust buffering mechanisms, and that consequent decanalization and associated gene-environment interactions could contribute to the pathogenesis of neurodevelopment disorders including schizophrenia and ASD (34, 35). Alternatively, the neocortex is more complex and thus more prone to disruptions than other brain areas, so that developing compensations for disruptions might be harder because of this inherent higher complexity.

Going beyond genetic associations, alterations in other cellular processes have begun to be found in ASD. Studying changes to physiology, gene expression, and the epigenetic states that contribute to ASD phenotype along with genetics, has begun to broaden our understanding of ASD and will eventually, in combination, lead to better methods of diagnosis, prognosis and even treatment of ASD. For the rest of this review, we focus on these mechanisms, briefly reviewing physiology and gene expression and focusing mostly on epigenetics, and in particular, the genome-wide studies of ASD published to date.

## The Physiology of Autism

Autism spectrum disorders are currently defined on the basis of behavioral observations only. However, evidence is growing rapidly that is beginning to define ASD in physiological terms (reviewed in Ref. (36–39)). If consolidated, such information may help to phenotypically define ASD, inform on prognosis and even help identify possible causes.

At the center of the ASD-associated physiological definition is the process of inflammation. Evidence for the association of inflammation with ASD comes from a relationship between familiar autoimmune disorders and ASD, discovery of a physiologically defined shift to a pro-inflammatory state (exemplified by cytokines) in the brain and blood in ASD, from animal models of ASD and from the positive response to immune suppressive medications (e.g., corticosteroids) in those affected by ASD (36–39). Affected immune cells include the microglia in specific brain regions (supported by post mortem and neuroimaging data) and cells of the innate and adaptive immune system in the blood.

Two other main physiological characteristics of ASD have been reported and reviewed: elevated levels of oxidative stress and mitochondrial dysfunction in the blood and brain (reviewed in Ref. (9, 37, 40)). Moreover, there are emerging links between inflammation, oxidative stress, and mitochondrial function and although the direction of associations are as yet unclear, a number of studies have found a relationship between the degree of disruption of these pathways and the severity of ASD (37). However, a lot more work needs to be done to test these associations, address the issue of causality and to use this knowledge to benefit those with ASD.

A further pathway implicated in ASD is intestinal dysbiosis as evidenced by a reduced microbiome complexity in ASD (reviewed in Ref. (38, 40–42)). However, again, issues of cause/effect and consistency of data need to be addressed before such data can be put to clinical use.

## Gene Expression and Autism

As the expression levels of a gene are largely influenced by the epigenetic state of its surrounding regulatory regions, it is first worth briefly reviewing genome-wide studies of gene expression in ASD. For a more extensive review, please refer to a paper written by Voineagu and colleagues (43). Below, we review the major findings from this publication and subsequent literature (44–49). Table 2 lists the samples, tissues, and major molecular pathways identified by these studies. The most commonly identified pathways have been inflammation and immunity (eight studies), neurodevelopment (four studies), synaptic function (four studies), steroid biosynthesis and metabolism (three studies), and circadian rhythm (two studies). Immune and inflammatory pathways have featured rarely in the results of genetic studies of ASD (for an exception, see Ref. (18)) but are no surprise in studies of expression because immune dysfunction in brain and blood have been well documented in ASD (38, 50, 51). “Neurodevelopment” is an expected pathway, as ASD are primarily a disorder of brain development. Steroid pathways, particularly those linked with androgens, may relate to the male predominance of ASD, but have also not been identified as common pathways disrupted by genetic change in ASD (52).

**Table 2:** **Summary of genome-wide studies of expression in ASD**.

Reference	Samples (cases/controls)^a^	Tissue source	Pathways identified
(53)	3/3^b^	LCLs	Neurodevelopment
(54)	49/12	PBLs	Immune and inflammatory response (mediated by NK cells), cytotoxicity
(55)	15/15	LCLs	Cell communication, immune and inflammatory response
(56)	20/–	LCLs	Steroid hormone metabolism
(57)	86/30	LCLs	Steroid hormone metabolism, circadian rhythm
(58)	52/27	PBLs	immune and inflammatory response (mediated by NK cells)
(59)	20/22	LCLs	Neurodevelopment, synaptic function (long-term potentiation)
(60)	10/23	CB, PFC, CN	Synaptic function
(61)	6/6	TC	Immune and inflammatory response
(62)	19/17	FC, TC, CB	Synaptic function, immune and inflammatory response
(46)	32/40	PFC, FC	Microglial function, immune response, neuronal activity
(63)	70/60	PBLs	Neurodevelopment; signaling; skeletal development
(48)	20/18	PBLs	Ribosome function, spliceosome function, mitochondrial, immune and inflammatory response, calcium signaling
(47)	170/115	PBLs	Neurotrophic signaling, notch signaling; synaptic function (long-term potentiation)
(45)	60/68	PBMCs	Immune and inflammatory response; hemoglobin metabolism
(49)	3/3^b^^,^ ^c^	LCLs	Neurodevelopment, skeletal development, gastrointestinal development, steroid hormone metabolism, circadian rhythm

*LCLs, lymphoblastoid cell lines; CB, cerebellum; PFC, prefrontal cortex; CN, cingulate nucleus; FC, frontal cortex; TC, temporal cortex*.

*^a^Some cases also had other syndromes such as Fragile X*.

*^b^Three discordant twin pairs, two with unaffected siblings*.

*^c^MicroRNAs analyzed. Adapted from Ref. (43)*.

## Epigenetics

Epigenetics describes the molecular factors that form complexes at regulatory regions of DNA to influence genetic activity without changing the primary DNA sequence. Such factors are usually inherited through mitosis but their meiotic (transgenerational) inheritance is controversial due to the expansive epigenetic remodeling that happens twice per generation – during gametogenesis and during very early embryonic development (64–66). Epigenetic change is initiated by sequence-specific proteins or RNAs. This results in the recruitment of epigenetic “writers” or transferases that add specific small molecules such as the methyl and acetyl groups to DNA or its packaging proteins – the histones. In turn, each locus recruits specific combinations of “readers” – molecules that bind modified DNA and histones. Finally, these readers can be removed by “erasers” such as demethylases or deacetylases. A further set of proteins – ATP-dependent epigenetic remodeling complexes – facilitate this process by loosening the ties between DNA and histones.

DNA methylation is the most widely studied and widely understood epigenetic mark and involves the covalent attachment of a methyl (CH_3_) molecule to the cytosine of a CpG dinucleotide (64, 65). In a minority of tissues and developmental time points, CpG can also be modified in other ways. Of relevance to this review, cytosines within CpGs can also be hydroxymethylated within the brain (67). DNA methylation can occur throughout the genome. The main regulatory regions of the genome, promoters and enhancers often contain a high density of CpG and an increase in methylation will usually correlate with a local, closed-chromatin structure. For many gene promoters, this is associated with silencing of the associated gene. For other regions, associations are context-dependent. For example, an open-chromatin structure could facilitate the establishment of a repressor or enhancer complex that could interact in *cis* or *trans* with a gene’s promoter.

Both genetic and environmental variation, and an interaction between the two, can influence epigenetic change (68, 69) (Figure 1). For example, variation in local DNA sequence may create or destroy the binding sequence for a sequence-specific protein or RNA. In addition, genetic variation in a gene encoding a protein with epigenetic function may result in aberrant activity of this protein which may affect the activity of all the genes that it regulates. Environmental factors are also known to influence epigenetic marks prenatally and throughout life, include smoking and stress (reviewed in Ref. (70, 71)). There are currently very few replicated environmental epigenetic biomarkers.

### Association, causation, and biomarkers

A statistically significant association of an epigenetic state with a disorder after its onset could mean one of two things: that the epigenetic state mediates the cause of, or results from, the disorder. Although the former is a more attractive conclusion, evidence must be accumulated that test this hypothesis. For example, if the same epigenetic state were present prior to onset of symptoms, ideally at birth, this would support the hypothesis. Furthermore, if the environmental cause was linked with both epigenetic state and the disease phenotype via an approach such as Mendelian randomization (72, 73), this would also support the hypothesis. It is also worth noting that epigenetic state, like all phenotypes, is influenced by a combination of environmental, stochastic, and genetic factors. Genetic factors predominate in about one fifth of loci (69, 74). Mechanisms behind such an interaction include the creation or destruction of a CpG site and alterations to the binding affinity of sequence-specific binding factors. Approaches such as Mendelian randomization, twin studies, and a focus on specific genes and environments can help to quantitate the environmental and genetic components of epigenetic state (73, 75–77). Factors such as gene-environment interaction (69) should also not be overlooked.

Epigenetic state can be used as a biomarker of disease risk, diagnosis, prognosis, and response to treatments (reviewed in Ref. (71)). Such biomarkers are already in use in the clinic for cancer; it is only a matter of time before replicated biomarkers are used for other diseases. The caveat for brain disorders, of course, is that the key tissues mediating symptoms are not readily available for biopsy. Therefore, readily available peripheral biomarkers, such as those identified in blood and buccal cells, must serve as surrogates for epigenetic changes, which have occurred in the brain. This is discussed in the following section.

### Epigenetics and autism

Epigenetic modification is increasingly thought to play a role in ASD, based on the findings discussed below. As mentioned previously, genes that play a role in epigenetic pathways constitute a sizable proportion of ASD candidate genes identified through genetic screens (21, 78, 79). In addition, evidence is mounting from human and animal studies that early life environment, including *in utero* and during early postnatal life, may play a role in ASD and other neurodevelopmental disorders (80, 81). Most studies have focused on the epigenetic mark of DNA CpG methylation. Effect sizes (mean methylation difference between cases and controls) have been relatively low, mostly less than 0.1 (10%). As a rough guide considering levels of technical accuracy, combined with the expected frequency of type 1 errors, >5% effect size is considered a reasonable effect size and >10% effect size would be a more ideal effect size with a higher likelihood of biological plausibility.

#### Can Studying Epigenetics Shed Light on Mechanisms and Biomarkers for ASD?

When we study disease-associated epigenetic marks such as DNA methylation we are usually looking for two things: (1) clues to the cause and mechanisms of a disorder and (2) biomarkers for its risk, diagnosis, or prognosis (71). Clues to the causal factors for a disease will come from cross-referencing with studies of the effect of specific environmental components on the epigenome from human or animal studies (71, 82). Clues to the mechanism will be through detailed analysis of gene networks and pathways and include systems-based approaches (82). As discussed earlier, with the former, we need to be mindful of issues such as reverse causation and genetic influences on epigenetics. With the latter, although desirable, there is no strict rule that a biomarker associates with a causal pathway.

Therefore with ASD, we have a number of options when planning epigenetic studies. We can search for (1) signatures of causative genetic or environmental factors; (2) clues to the physiological mechanisms predisposing or resulting from the onset of ASD; (3) biomarkers that will contribute toward a better prediction of risk prior for ASD prior to diagnosis; (4) biomarkers that will aid in diagnosis, including endophenotyping and (5) biomarkers that may help in predicting symptom severity or diversity. However, before we do this, there are a number of considerations we need to make.

#### Tissues used to Study ASD-Associated Epigenetic States

ASD is thought to be primarily a disorder of neurodevelopment involving multiple regions of the brain including frontal, temporal, and occipital lobe cortices and the cerebellum (83–85) (Figure 1). Post mortem studies of genome-wide expression and DNA methylation have analyzed selected brain regions obtained from biobanks. Despite notable efforts (86), brain biobanks are rare, especially those with samples from children (87). As brain tissue cannot be collected from live individuals, there are two ways to address this issue: to establish more brain banks (87) and to use readily obtainable peripheral tissues. The latter deserves some discussion here. Each human tissue contains genomic regulatory regions in which epigenetic state is tissue-specific, regions whose epigenetic state is shared with a subset of other tissues and regions whose epigenetic state is common to all tissues (88–90). The Human Epigenome Project (91) and other epigenomic data repositories (e.g., Ref. (92)) can be used to distinguish between each of these classes. However, only datasets obtained from samples from those with a specific disorder such as ASD can truly determine between-tissue correlations for that specific disorder, as disease state is likely to have some tissue specificity. Nevertheless, the physiological evidence, summarized above, of immune, mitochondrial, and oxidative stress dysfunction in ASD imply that blood and other easy-to-obtain peripheral tissues could be studied in their own right. It has also been argued that environmental components of disease may leave soma-wide footprints on the epigenome (93). But can we go one step further in ASD? Can peripheral tissues be used as proxies for epigenetic state in the brain? This is a complex issue. High correlations have been found between methylation levels of specific genes in blood and brain regions in neurodevelopmental disorders and controls, for example, *COMT* (in multiple brain regions in schizophrenia (94)), *OXTR* (in temporal cortex in ASD (95)) and *HGC9* (in prefrontal cortex, occipital cortex, corpus callosum in bipolar disorder (96)). Furthermore, 50% of a set of schizophrenia candidate genes and gene ontologies were found to be expressed in both PBLs and prefrontal cortex (97) and there is a significant correlation between DNA methylation in the blood, and cerebral cortex and cerebellum (90).

Finally, genome-wide methylation profile in buccal epithelium shows a higher similarity than blood in comparison with the brain (98, 99), most likely because of a shared ectodermal origin. Thus, if an environmental or stochastic event occurred in this lineage in early embryogenesis, theoretically, effects could be seen in both tissues. Epigenetic changes within buccal epithelium have also been used as a proxy for the effects of early life environment on the brain (100, 101). Peripheral tissues also have the advantage that they can be sampled repeatedly and even retrospectively, in the case of dried heel-prick blood spots taken just after birth (102).

#### Methylation Analysis of ASD Candidate Genes

Although they are being superseded by genome-wide approaches (Section “Epigenome-Wide Analysis of ASD”), candidate gene approaches to the study of DNA methylation in ASD are still valuable. They can afford a more detailed analysis of the epigenetic state of a gene for which there is circumstantial evidence for its dysregulation in ASD and allow comparison with RNA and protein expression. Below, we review the small number of genes identified by such approaches. Brain regions previously shown to be associated with ASD are usually examined, such as temporal cortex (responsible for processing auditory input, high level visual processing, language comprehension, and emotion association), cerebellum (coordinates voluntary movements, posture, and balance), occipital lobe cortex (visual processing), dorsolateral prefrontal cortex (working memory and executive function including the regulation of thinking and action), anterior prefrontal cortex (cognitive function), and anterior cingulate cortex (error detection and conflict monitoring).

##### Oxytocin receptor

The oxytocin receptor (OXTR) is a G-protein coupled receptor for the peptide hormone and neurotransmitter oxytocin. It plays a role in anxiety, social memory and recognition, sexual and aggressive behaviors, and maternal-offspring bonding. *OXTR* has a score of 3 (“suggestive evidence”) in the SFARI ASD gene database and a recent meta-analysis found significant associations of four *OXTR* SNPs with ASD (103). The first evidence of an association between *OXTR* methylation and ASD came from a study of two siblings with ASD, only one of which had an *OXTR* deletion (95). Compared to his father, the sibling without the *OXTR* deletion had differential DNA methylation in peripheral blood leucocytes (PBLs) in a region of the *OXTR* promoter previously shown to regulate expression. The highest difference in methylation at a single CpG was 37.5%. Further analysis of peripheral blood mononuclear cells (PBMCs) in 20 sex-matched ASD cases and controls showed 23% higher DNA methylation in ASD at specific CpGs. When males and females were analyzed separately, the magnitude of these differences was much larger in males (39% higher in ASD), underlining the need to test for sex effects in such studies. The study went further by comparing levels of DNA methylation in temporal cortex from ten ASD cases and age- and sex-matched controls. Similar sex-specific differences were found in temporal cortex and PBLs, with up to 41.6% higher methylation at specific CpG sites in males with ASD (95). Higher methylation levels in males correlated with lower *OXTR* expression in temporal cortex. Caution must be taken with interpretation of this data because blood and brain samples did not come from the same individuals. Linking DNA methylation with a particular ASD-associated endophenotype, Jack and colleagues found a strong correlation between *OXTR* promoter methylation in PBMCs and perception of animacy (purposeful movement) of an inanimate object, with correlated neural responses in two brain regions measured by functional magnetic resonance imaging (fMRI) (104). The inter-individual differences in DNA methylation were around 30%, well within the range of biological plausibility. Associations have also been found between DNA methylation of *OXTR* and traits with phenotypes overlapping with ASD, such as social anxiety disorder (105), and callous, unemotional traits (106, 107), reviewed in Ref. (108).

##### Glutamate decarboxylase 1

Glutamate decarboxylase 1 (*GAD1*, also known as *GAD67*) encodes an enzyme that catalyzes the production of GABA (gamma-aminobutyric acid), the chief inhibitory neurotransmitter, from glutamate. There is little evidence linking genetic variation in *GAD1* with ASD. It has an entry but no score in the SFARI ASD gene database (13). However, *GAD1* expression is disrupted in Purkinje neurons in the cerebellum from ASD patients (109, 110). Furthermore, complete loss (111) or haploinsufficiency (112) of *Gad1* in mice leads to deficits in learning and social behavior. A recent study found an increase in DNA hydroxymethylation (of ~3%) at the *GAD1* promoter in cerebella from ASD patients using two different methods (113). This change was accompanied by increased binding of the methyl CpG binding protein 2 (MeCP2), which plays a role in gene silencing. This illustrates that a gene may be associated with ASD through epigenetic, but not genetic, change. This is important because *Gad1* expression was reduced by prenatal exposure to the anticonvulsant valproate in rats (114) and by a prenatal immune challenge in mice (115, 116). Both types of exposures have been linked with an elevated risk for ASD (117, 118). Taken together, current evidence suggests that *GAD1* is a good candidate for a gene that could be epigenetically mis-regulated by *in utero* environment, predisposing to ASD.

##### Engrailed-2

Engrailed-2 (*EN2*), which encodes a homeodomain-containing protein, has been implicated in the control of pattern formation during neurodevelopment (119). *EN2* has a score of 4 (“minimal evidence”) in the SFARI ASD gene database (13). The *EN2* promoter had increased levels of DNA methylation in ASD cerebella compared to asymptomatic controls (120). Two different techniques showed significant differences of 10–20% methylation. A similar relationship was later found with hydroxymethylation (121). DNA methylation was also positively correlated with levels of *EN2* RNA and protein levels and negatively correlated with levels of repressive epigenetic marks in the same tissue (120, 121).

##### Reelin

Reelin (RELN) is a secreted extracellular matrix glycoprotein involved in neuronal migration and positioning in the developing brain and modulates synaptic plasticity in adult brain. It has a score of 2 (“strong candidate”) in the SFARI ASD gene database (13). Similar to *GAD1*, a recent study found an increase in levels of DNA methylation and a decrease in hydoxymethylation at the *RELN* promoter in cerebella from ASD patients, accompanied by an increased binding of MeCP2 (113). Lower levels of *RELN* mRNA were found in frontal cortex and cerebellum in individuals with ASD (113, 122) and lower levels of RELN protein have been found in the brain (122, 123) and plasma (124) of those with ASD.

##### MECP2

*MECP2* plays a role in gene silencing. Mutations in *MECP2* cause Rett syndrome, which has overlapping phenotypes with ASD (125). It is referred to in the SFARI ASD gene database as a “syndromic gene” (13). Abnormal levels of *MECP2* transcript and protein together with atypical splice variants have been found in frontal cortex from patients with ASD (126). In a follow-up study, the same group found lower levels of *MECP2* expression in frontal cortex in 11/14 ASD cases compared to age-matched controls and an increased level (up to 12%) of DNA methylation at the *MECP2* promoter in males, which negatively correlated with levels of MECP2 protein (127). In a third study, the investigators focused on a transition region of the *MECP2* promoter, separating regions of female-specific from sex-independent methylation in frontal cortex (128). DNA from male ASD patients showed increased levels of DNA methylation (~10%) while females showed some abnormal methylation. No evidence was found for general defects in X inactivation in blood and brain prompting the conclusion that aberrant *MECP2* methylation in ASD brain DNA is due to locus-specific rather than global X chromosome methylation changes. Of note, a mouse model of ASD found an effect of maternal immune activation on *Mecp2* methylation (129), again showing that ASD-associated changes in methylation can arise from adverse *in utero* environments.

##### Summary of candidate gene analyses

The evidence for the association of each of the above five candidate genes with ASD is summarized in Table 3. All candidates have evidence of association with ASD beyond genetics and DNA methylation. Plausibility is increased by effect sizes >10% (*OXTR, EN2, MECP2*), from associations with expression (all genes), protein levels (*EN2, RELN, MECP2*). For all candidates, methylation differences were found in different brain regions, but only *OXTR* has shown differences in a peripheral tissue (blood). All four candidates are worthy of further attempts at replication, ideally in multiple brain regions and multiple tissues.

**Table 3:** **Summary of evidence for potential ASD-specific methylation biomarkers**.

Gene	Genetic evidence	Methylation reference	Diagnostic method	Tissue	Samples^a^	Largest effect size^b^	Expression	Protein	Other data
*OXTR*	Weak	(95)	DSM-IV, ADI-R	PBLs	20/20	+23%	No	No	Endophenotype^d^
					10/10^c^	+38.9%	
				Temporal cortex	10/10^c^	+41.6%^c^	yes	No	
*GAD1*	Weak	(113)	Not given	Cerebellum	10/10	+3%^e^	Yes^f^	No	Animal models, MECP2 binding
*EN2*	Minimal	(120)	DSM-IV	Cerebral cortex	13/13	+10–20%^g^	Yes	Yes	
*RELN*	Strong	(113)	Not given	Cerebellum	10/10	Not quantifiable	Yes	Yes	MECP2 binding
*MECP2*	Syndromic	(127, 128)	ADI-R, ADOS	Frontal cortex	14/14	+12%, +10%^h^	Yes	Yes	Animal model

*For abbreviations and references see the main text*.

*^a^Cases/controls*.

*^b^% methylation difference, cases minus controls*.

*^c^Males only*.

*^d^Methylation correlated to endophenotype*.

*^e^Hydoxymethylation only*.

*^f^In Purkinje neurons and cerebellum*.

*^g^Methylation and hydroxymethylation*.

*^h^Two separate sets of *MECP2* CpG sites regions measured*.

#### Epigenome-Wide Analysis of ASD

Seven recent studies have gone beyond candidate genes to study levels of DNA methylation on a genome-wide scale, commonly termed as epigenome-wide association studies (EWAS) (130–136). By “genome-wide,” we refer to methods that query epigenetic state on all human chromosomes, although not necessarily at every CpG in the genome. Below, we briefly review the different EWAS methods used in these epigenetic studies and discuss the major issues associated with their interpretation.

Epigenome-wide association studies methods fall into two major classes: those based on the affinity of a molecule for methylated cytosine and those based on the sequence difference resulting from conversion of only non-methylated cytosine to uracil using sodium bisulfite (137). Each method comes with its own strengths and weaknesses. Affinity-based methods analyze the whole genome but have a bias toward CG-rich regions, have a relatively low resolution (not individual nucleotides) and are considered only semi-quantitative. The fractions of the (epi)genome they isolate can be analyzed using microarrays of by high-throughput sequencing. Examples reviewed below include methylated CpG island recovery assay (MIRA) (134, 138, 139) and methylated DNA immunoprecipitation (MeDIP) (135, 140). The most commonly used array-based method is the Infinium BeadChip array which targets specific CpG sites using bead attached probes complementary to specific genomic regions which assay specific CpGs at one end of the probe (141). HM27 arrays, now no longer available, contain probes for 27,578 CpGs covering 14,469 genes, with enrichment for CpG islands and proximal promoter regions and for genes associated with cancer (141–144). HM450 (also known as “450K”) arrays contain probes for 482,421 (or 1.7%) of the 28 million CpG sites genome-wide. Probes include most of those on HM27 arrays and an additional set targeted to those regions of the genome judged to be of regulatory importance by an expert panel. Such regions include promoters, enhancers, CpG island “shores” (2 kb either side of CpG islands) and “shelves” (2 kb either side of shores) together with intragenic and intergenic regions. An advantage of HM450 arrays is that they are considered quantitative and their main disadvantage is their low genomic coverage, especially in intergenic regions. This means that they could miss some differentially methylated regions (DMRs) picked up by affinity-based methods. Nevertheless, they are the current method of choice for most genome-wide studies of DNA methylation in humans mainly because of their relatively low cost and their quantitative nature. The ultimate resolution for genome-wide studies is bisulfite sequencing of the entire genome, which is currently cost-prohibitive for most studies, although methods have been developed to sequence functionally important regions of the genome (reviewed in Ref. (137)).

There are a number of “best practices” when it comes to planning, conducting, and analyzing genome-wide methylation analysis that will assist in the interpretation of the studies reviewed below (reviewed in Refs. (137, 145–148)). First, false positives should be minimized by adjusting for genome-wide multiple testing and the use of a false discovery rate (FDR) cut-off of adjusted *p* < 0.05 or <0.1. False positive rate can be further reduced by selecting only those probes or regions clustered within genomic space; this is especially important for HM450 arrays that rely on the hybridization of single probes within a given region. Further recommended options to decrease the false positive rate include validation of top differentially methylated probes (DMPs) or DMRs using an independent, locus-specific method (149–151) on the same samples and using a cut-off for effect size as mentioned above.

Even when these conditions are satisfied, findings need to be replicated in independent cohorts. While the use of a different method of genome-wide analysis is desirable, comparison between different platforms can be difficult for reasons including differences in genomic coverage and resolution. One further consideration is the functional relevance of disease-associated differences in DNA methylation. The “best case” scenario is when methylation correlates with expression of a nearby gene that has relevance for the specific disorder, but such associations are often not found or not looked for. Having said that, as discussed earlier, functional relevance is not an absolute requirement for an epigenetic biomarker.

At the design stage, accurate phenotyping and study power are important issues to consider. As with other “omics” studies, bigger is usually better, although the proportion of variance in phenotype explained by single epigenetic variants appears to be much larger than genetic variants (e.g., Ref. (152, 153)). Power has been difficult to calculate in genome-wide studies of DNA methylation because every CpG or region has a different between-subject variance. Typical published sample sizes have been growing from two to three and even four digit numbers over the past few years and again, necessary sample size is dependent on effect size (137). Twin studies, recommended as the place to start for epigenome-wide analysis, can withstand smaller sample sizes because within-pair analysis controls for sex, age, parents, family environment, and genetics (more in identical than fraternal twins) (75, 137).

At the analysis stage, potential confounders, such as age, sex, genetic factors such as ethnicity, biological (e.g., heterogeneity of tissues such as blood), and technical variation, need to be queried. Issues of cause vs effect and which tissue to analyze have already been discussed.

Below we review, to our knowledge, all the genome-wide studies of DNA methylation conducted on ASD samples (summarized in Table 4), covering first the studies on brain tissue and then peripheral tissue.

**Table 4:** **Summary of genome-wide studies of methylation in ASD**.

Reference	Samples^a^	Tissue	Participant age (years)	Diagnostic method	Method of analysis	DMR/DMP analysis^b^	Effect size cut off ^c^	Adjustment for multiple testing	Validation^d^	Expression data^e^
(131)	9/9^f^	Occipital cortex	1–60	DSM-IV, ADOS, and/or ADI-R	HM27	DMP	No	Yes	No^g^	Yes
		Cerebellar hemispheric cortex	
(132)	12/21	Prefrontal cortex	17–35	ADI-R and/or ADOS	HM450	DMR	No	Yes	No	No
	16/21	Temporal cortex	21–40						Yes^h^	
	13/21	Cerebellum	14–17						No^i^	
(133)	11/11	Anterior cingulate gyrus	16–51	ADI-R	HM450	DMP	>5% difference	Yes	Yes	Yes
	12/12	Prefrontal cortex	
(134)	3^f^, 10	LCL	2–19	ADI-R	MIRA	DMR	No	Yes	Yes	Yes
(135)	5/5	PBLs	6–12	DSM-IV, MINI instrument	MeDIP	DMR	>1.5-fold change	No	Yes	Yes
(136)	6^j^	PBLs	15	CAST	HM27	DMP	No	No	No	No
	16/22								Yes
	6/10^k^								No	
	50^l^^,^ ^m^								No	
(130)	47/48	Buccals	1–28	Not stated	HM450	DMR	No	Unclear^n^	Yes	No

*For abbreviations see the main text*.

*^a^Cases/controls*.

*^b^Analysis based on probes (single CpGs) or regions (groups of CpGs)*.

*^c^Use of a threshold of minimal effect size (% methylation)*.

*^d^Using a locus-specific method*.

*^e^Attempted to correlate methylation with expression at DMRs*.

*^f^Only males*.

*^g^Validation process was done but findings were not validated*.

*^h^Some findings were validated in temporal cortex and cerebellum samples*.

*^i^Findings were not validated in the replication study*.

*^j^ASD-discordant MZ twins*.

*^k^Sporadic ASD/familial ASD*.

*^l^For regression of CAST scores and methylation*.

*^m^Total number of MZ twin pairs*.

*^n^DMR identification was confirmed by re-running “bump-hunting” four times*.

##### Genome-wide analyses of DNA methylation in the brain in ASD

Using DNA from the cerebellar cortex and occipital cortex from nine men with ASD (diagnosed using DSM-IV, ADOS, and/or ADI-R) and nine age-matched controls, Ginsberg and colleagues used HM27 arrays to identify ASD-specific probes at an FDR of 0.05 (131). No significant DMPs or differentially methylated gene networks were found. An array-based genome-wide gene expression analysis produced 876 genes differentially expressed between autistic and control brains at an FDR of 0.05. Ontology analysis showed down-regulation of genes involved with mitochondrial oxidative phosphorylation and protein translation. The former but not the latter have been identified in physiological studies of ASD. The authors also found differential expression of genes involved in synapse formation and other brain functions, some of which had been discovered using genetic analysis. Breaking down ASD into sub-phenotypes, two gene modules were significantly associated with social interaction and one gene module with stereotyped and repetitive behavior. Immune response was a common feature of these modules.

Ladd-Acosta and colleagues analyzed DNA methylation, using Infinium HM450 arrays, in the dorsolateral prefrontal cortex (*n* = 12), temporal cortex (*n* = 16), and cerebellum (*n* = 13) from individuals with ASD, diagnosed using ADI-R and/or ADOS, and 21 control brains matched for age, sex, and post-mortem interval (132). After removal of SNP-containing probes, they applied a further cut-off for those probes that were clustered in genomic space (adjacent probes within <500 bp). FDR < 0.1 was used to produce over 1000 DMRs for each tissue.

The authors focused on the three DMRs with the lowest adjusted *p*-values. The first, found in temporal cortex, was within a possible bidirectional promoter for the tetraspanin 32 (*TSPAN32*) gene and the uncharacterized transcript *C11orf21* on chromosome 11. This DMR extended into the body of *C11orf21*, spanned 1.5 kb and 26 HM450 probe CpGs and was on average 6.6% less methylated in ASD. “Replication,” performed using HM450 data from specific probe CpGs on the other two tissues, showed evidence for this DMR in cerebral cortex. TSPAN32 is a tumor suppressor and is involved in hematopoietic cell function and cellular immunity (154). *TSPAN32* and *C11orf21* lie within a region of chromosome 11 rich in imprinted genes including *IGF2* and *H19*. However, current data suggest that *TSAPN32* is biallelically expressed in most tissues and species, with the exception of day 9.5 placenta in mouse (155) and human placental trophoblasts (156), where it is expressed from the maternal allele only. No studies of *TSPAN32* or *C11orf21* imprinting or function have been performed in brain tissue. Outside the placenta, the brain is one of the most frequently imprinted, but least-studied, tissues (157). Imprinted loci are particularly susceptible to environmental influence due to their haploid expression.

A second temporal cortex DMR was located in the 3′ untranslated region (3′UTR) of the proline-rich transmembrane protein 1 (*PRRT1*) gene close to the MHC region on human chromosome 6. This DMP encompassed a few hundred kb, 33 HM450 probes and was on average 7.8% less methylated in ASD brains. Fourteen individual probes were replicated as above, in prefrontal cortex and cerebellum. Little is known about *PRRT1* function in mammals.

A third temporal cortex DMR was located in a region ~3.5 kb upstream of the zinc finger gene *ZFP57*, also close to the MHC region on chromosome 6. This DMR also encompassed a few hundred bp, ~12 HM450 probe CpGs and was, on average, 13.9% more methylated in ASD cases. Replication showed DMRs in temporal cortex and cerebellum in females only. ZFP57 is a transcription factor that binds preferentially to methylated DNA within imprinted DMRs and in doing so, maintains DNA methylation and genomic imprinting during development (158). The DMR also maps to a known regulatory element of *ZFP57* (159) and the same region was also the strongest DMR in a study of the effects of maternal folate intake on cells from the innate and adaptive immune systems (160). As folate intake and metabolism have been implicated, but not proven, as a potential contributor to ASD risk (161–163), this gene could be a possible mediator between environment, genetics, and epigenetics in ASD.

A further DMR was found in cerebellum using the above methods within a 1 kb region around the promoter region of the succinate dehydrogenase complex subunit A flavoprotein pseudogene 3 (*SDHAP3*) on chromosome 5. This DMP comprised ~10 HM450 probe CpGs and was, on average, 15.8% more methylated in ASD cases. An attempt to replicate these findings in other brain tissues showed no significant DMPs, although a general trend toward a similar magnitude of DNA methylation was seen. Unlike the other ASD DMPs, evidence was found for a causal relationship between local copy number variation and DNA methylation at the *SDHAP3* DMR, but the authors argued that this still implicated the gene in the etiology of ASD. Little is known about the function of SDHAP3 other than it has coding and non-coding transcripts. Mutations in other succinate dehydrogenase (electron transport chain Complex II) components cause a number of human metabolic disorders (164), possibly linking this DMR with mitochondrial dysfunction seen in ASD.

Ladd-Acosta and colleagues also investigated the possibility that differences in cellular heterogeneity between ASD and control brains influenced methylation levels in their data (132). Using published data on cell type-specific methylation in the brain, the authors found no evidence for an effect of cellular heterogeneity on methylation levels within their DMRs. The authors did not perform pathway analysis of their DMRs.

Nardone and colleagues analyzed DNA methylation, also using Infinium HM450 arrays, in the anterior cingulate gyrus and prefrontal cortex from 11 and 12 matched ASD (diagnosed using ADI-R) and control pairs respectively (133). They selected DMPs using an FDR <0.05, and a cut-off of at least 5% difference in methylation between cases and controls. This analysis yielded >5000 DMPs for each tissue. Both sets of DMPs were enriched in CpG island shelves, intergenic regions and gene bodies and depleted in CpG islands and promoters. Such patterns are found in regions more likely to change over time during prenatal development and aging ((165) and references therein) and including regions whose methylation changes significantly during the development of the prefrontal cortex in mice and humans (166). In addition, the two brain regions studied were significantly more epigenetically similar in samples from ASD than those from controls. The authors concluded that these findings were the manifestation of a disruption to methylation-associated neurodevelopment. Gene pathway analysis showed enrichment in DMP-associated genes for immune-related biological processes such as leukocyte migration, cytokine-mediated signaling pathways and inflammatory response and to a lesser extent, synaptic transmission. DMPs showed little overlap with the SFARI gene database but did overlap with the dataset of ASD-associated differences in expression from an adjacent region of the dorsolateral prefrontal cortex (BA9 compared to BA10 studied by Nardone and colleagues) from an independent transcriptomic analysis (62). In addition, genes associated with DMPs that had higher levels of DNA methylation in ASD samples overlapped significantly with genes that were under-expressed in ASD. The authors suggested that the dearth of immune-related genes in genetic studies of ASD implied that such genes were more likely to be influenced by epigenetic than by genetic change. This agrees with the findings of Ginsberg and colleagues above (131). The authors observed that many of the immune-related genes they identified are also associated with microglial function. Microglia act as macrophages in the brain, with roles in synaptic pruning and in guiding cell fate during brain development (167). The authors ranked gene-associated DMPs by the number of DMPs associated with each gene (as opposed to clustering of probes in absolute genomic space). None of the highest-ranked genes had previously been associated with genetic studies of ASD. The highest-ranked cluster of CpGs (20 probes, each one located within 500 bp of adjacent probe CpGs) lay in almost exactly the same region of *TSPAN32/C11orf21* as that identified by Ladd-Acosta and colleagues (132). Like the former study, probes were on average 8% less methylated in ASD brains. Seven of these probes were validated using pyrosequencing. Of note, three prefrontal cortex-specific DMPs (average 9% less methylation in ASD), all within 1 kb at the 3′UTR of the *PRRT1* gene coincided with one of the top four DMRs identified previously in prefrontal cortex and cerebellum (132). One of these DMPs was also found in anterior cingulate gyrus. A single anterior cingulate cortex DMP was also found in *ZFP57* – the same gene as that associated with a previously identified DMR (132), although in a different location within the gene.

##### Genome-wide analysis of DNA methylation in peripheral tissues in ASD

The first study of genome-wide DNA methylation in ASD on peripheral tissue analyzed DNA from B-cell derived lymphoblastoid cell lines (LCLs) from three pairs of twins discordant for severity of ASD (134). Although one twin from each pair was diagnosed with ASD using ADI-R, all co-twins had ASD-like symptoms, albeit not severe enough to meet diagnostic criteria. Investigators used the MIRA method of affinity capture combined with CpG island microarrays containing ~12,000 CpG island promoters. Using an FDR cut-off of <0.1, 73 CpG islands were significantly differentially methylated within all three pairs. ASD-associated genes were enriched for genes involved in neurological disease, nervous system development/function, cellular assembly/function and embryonic function. Treating both twins from each pair as “broadly autistic” and comparing both with unaffected siblings resulted in 201 differentially methylated CpG islands. ASD-associated networks included nervous system development/function and neurological disease, as for the first analysis, together with control of transcription, and cell death. All further analyses were performed with this dataset. Cross-referencing with genes differentially expressed in the same samples (53) showed that all but one of the genes had high inverse correlations between methylation and expression. Network analysis of these genes highlighted the same pathways as those found with methylation alone, with the addition of inflammation, digestion and steroid biosynthesis (134). In the promoter of the B-cell CLL/lymphoma 2 (*BCL2*) gene, similar levels of methylation were found in undiagnosed twins and siblings (11.4 and 9.5%, respectively, validated using bisulfite sequencing), with ASD twins having around 15% higher methylation than the two types of controls. In the retinoic acid related orphan receptor alpha (*RORA*) promoter, qualitative analysis, using methylation-specific PCR, confirmed higher levels of DNA methylation in twins with ASD compared to all controls. Investigators then presented evidence for a role of DNA methylation in the expression of both *BCL2* and *RORA* and that levels of RORA protein are reduced in cerebellum and frontal cortex in ASD. RORA is a nuclear steroid receptor linked to transcriptional activation of genes involved with neurodevelopment, circadian rhythm, and protects against the effects of inflammation and stress (168). It had not previously been associated with ASD, although other studies have suggested a role of RORA in the environmental causes and the sex bias observed in ASD (169). BCL2 is a major player in cell death pathways and reduced levels of expression have been found in lymphoblasts (170) and frontal cortex (171) of those with ASD compared to controls.

Wang and colleagues analyzed DNA methylation, using MeDIP and promoter arrays, in PBLs from five children with DSM-IV-diagnosed ASD and five age- and sex-matched controls (135). Over 200 genes were significantly differentially methylated using a combination of an unadjusted *p* < 0.05 and at least 1.5-fold difference between ASD cases and controls. Thirteen of these genes were significantly differentially methylated in all five pairs. Four of these genes were selected for locus-specific validation using bisulfite sequencing but only enolase 2 (*ENO2*, also known as neuron-specific enolase and expressed in developing neurons (172)) was validated. This shows the importance of validation to decrease Type 1 errors. *ENO2* promoter methylation was 28.1% higher in ASD cases compared to controls. Of a total of 131 additional case-control pairs, only 19 (14.5%) of pairs showed differences of a similar magnitude, which were negatively correlated with *ENO2* expression. Therefore, with a larger sample size, *ENO2* did not replicate as a potential ASD-specific biomarker. No other associations between ASD and *ENO2* have been found previously or since. It is also worth noting that the promoter of brain-derived neurotrophic factor (*BDNF*) was also significantly differentially methylated in this study, with methylation levels differing between four out of five case-control pairs. Multiple studies have identified significantly different levels of BDNF protein in the blood of those with ASD compared to controls (173–177). However, no other studies have identified *BDNF* as differentially methylated in ASD.

Using PBLs from 50 pairs of monozygotic (MZ) twins, Wong and colleagues used Infinium HM27 arrays to identify specific differentially methylated CpG sites associated with ASD (136). Although authors referred to their differentially methylated probes as “DMRs,” strictly speaking, they were DMPs and this is how we will refer to them here. The authors used two major analytical approaches to identifying ASD-associated DMPs: within-pair and case-control analysis. CAST scores of >15/31 were used to assess whether a child was at risk of ASD.

In the within-pair analysis of six pairs of ASD-discordant MZ twins, a ranked list of top 50 DMPs were generated from six MZ twin pairs by combining significance (*t*-test unadjusted *p*-value) and effect size (methylation difference between cases and controls). The top DMP was located in the promoter of the nuclear transcription factor Y gamma (*NFYC*) gene with ASD cases showing an average 8% higher methylated compared to control co-twins. Other top co-twin DMPs included probes associated with the dual-specificity phosphatase 2 (*DUSP2*) gene, with 5% lower methylation on average in ASD co-twins. This gene had previously been identified as a target of microRNAs mis-regulated in ASD (49). In a second within-pair analysis, this time analyzing each pair separately, the authors found several DMPs that were common across two or more ASD-discordant twin pairs. These included the heme-containing peroxidase *PXDN* gene, with 24% higher methylation on average in the ASD-co-twin in two pairs, and the unannotated transcript *C110rf1*, with 29% higher methylation on average in the ASD-co-twin in two pairs.

A case-control analysis (*n* = 16 and 22, respectively) found DMPs within an intron of the unannotated transcript *MGC3207* (on average, 24% less methylated in ASD cases), within the putative promoter of the olfactory receptor gene *OR2L13* (on average, 18% more methylated in ASD cases) and within an intron of the transcript of unknown function *FAM181A* (also known as *C14orf152*; on average, 16% less methylated in ASD cases). Findings from the first two probes were validated by bisulfite pyrosequencing. The authors expressed caution with interpretation, noting that effects of local DNA sequence could be influencing methylation levels at these two probes, but ruled this unlikely at *MGC3207* because they could find no obvious local polymorphic sequence variant. Of note, the *OR2L13* expression is dysregulated in Parkinson’s disease (178). *FAM181A* was hypermethylated in peripheral blood from adults with asthma (179) and in peripheral blood from infants exposed to asthma during pregnancy (180). In one study, fathers with asthma were at higher risk of having a child with ASD (181).

To identify any epigenetic differences between sporadic (one twin of each pair with ASD, *n* = 6) and familial (both twins in each pair with ASD, *n* = 10), comparisons were made of ASD individuals between the two groups. The top DMP from this comparison was located upstream of the transcript of unknown function *C19orf33* (on average, 12% less methylated in individuals with sporadic ASD). Other significant subtype-specific DMPs were located within the methyl-binding domain gene *MBD4*, the autism susceptibility candidate 2 gene *AUTS2*, and the microtubule-associated protein gene *MAP2*, all previously associated with ASD (13). Both *AUTS2* and *MAP2* play a role in neurodevelopment.

Wong and colleagues also analyzed probes associated with the autistic trait scores of social, repetitive behaviors and interests, and communication. DNA methylation at multiple CpG sites were found to be correlated with CAST scores, including one in the putative promoter of the neurexin 1 (*NRXN1*) gene previously associated with ASD (13).

In addition, a small number of genes appeared in more than one of the above analysis. For example, *NFYC* and transmembrane phosphoprotein *PTPRCAP* genes contained top DMPs in both within-pair and case-control analyses.

Berko and colleagues analyzed DNA methylation using HM450 arrays and DNA from buccal epithelium from 47 cases of ASD (diagnostic tool not described) and 48 unaffected controls (130). Care was taken to exclude probes associated with known CNVs or SNVs. The authors also used an analytical approach which incorporated information on clustering of potential DMPs and adjustment for technical (e.g., microarray chip) and biological (e.g., age, sex) variation (182). This approach yielded nine ASD-specific DMRs, whose associated genes were all also expressed in the brain and six from nine associated with synaptic transmission, although none were previously identified as ASD-specific from genetic studies (13). One DMR, however, present within the *OR2L13* putative gene promoter, contained the ASD-specific DMP identified in the case-control analysis of peripheral blood (136). It was on average 8% lower in individuals with ASD but the direction of effect was opposite to the former study. *OR2L13* is upregulated in frontal cortex and downregulated in frontal cortex from individuals with ASD (62). DMRs at *OR2L13* and *FAM134B* were validated using bisulfite high-throughput sequencing. No significantly differentially methylated gene networks were identified. However, from their DMP data, the authors concluded that the same pathway, synaptic transmission, could be affected by genetic and epigenetic change in ASD and stressed the importance of combined genetic and epigenetic analysis.

## Strengths and Weaknesses of the Genome-Wide Studies of DNA Methylation in ASD

First, the low numbers of cases and controls in all studies are striking and way below the ideal of >100. Replication of potential ASD-specific methylation biomarkers must be attempted in much larger sample numbers. Next, an extremely diverse variety of biological samples were used in the different studies. Although the identification of the same gene in different tissues, methods and studies (namely *OR2L13*, *PRRT1*, *ZFP57*, and *TSPAN32*/*C11orf21*) suggest potential ASD-specific methylation biomarkers worthy of replication, this replication would also need to be within-tissue in future studies. Age of participants is also an issue of concern because it is possible that ASD-discordant pairs may grow more epigenetically discordant with time, meaning that DMPs/DMRs could either be age-related stochastic/environmental epigenetic changes or a simple effect of autism rather than on the causal pathway. Variation in the method of ASD diagnosis is also a major issue because each set of DMPs/DMRs is, to a large extent, specific for each diagnostic tool. Future studies should attempt to use only the most reliable method, i.e., DSM-V (183). Heterogeneity of analysis platform also clouds comparison between studies, for example some CpGs or regions will not be represented on all platforms. In addition, some methods (e.g., HM27, HM450) use probes, whereas others (MIRA, MeDIP) work with regions. Again, one or two core platforms, e.g., array and sequence-based to control for method-specific biases, would be ideal for all future studies. An ideal genome-wide methylation analysis would involve a focus on contiguous regions rather than single CpGs (probes) or annotated genes (131), adjustment for multiple testing, the use of a minimal effect size cut-off, validation with an independent method in the same samples or different samples from the same study population and replication in independent cohorts. As mentioned above, four genes fulfill the final criteria, but no one study fulfils all. Finally, only four studies looked for a relationship between methylation and expression for top DMPs/DMRs (131–135). In conclusion, although these studies identify a small number of potential ASD-specific biomarkers requiring replication, future studies should focus on larger numbers, harmonization of ASD diagnosis, validation, replication and other “best practices” such as adjustment for multiple testing.

## Synthesis

Despite their weaknesses, the seven recent studies of DNA methylation in ASD provide some useful insights into its etiology. Firstly, a small number of replicated, potential methylation biomarkers for ASD have emerged. For *PRRT1*, lower methylation (−9%) in a DMR 3′ UTR in temporal cortex and cerebellum (132) was replicated by lower methylation (−7.8%) in the same region in prefrontal cortex using the same platform (133). For a region upstream of *ZFP57*, higher methylation (+8.9%) in a DMR in temporal cortex (132) was replicated by higher methylation (+9.4%) in cingulate cortex also using the same platform (133). For *TSPAN32/C11orf21*, lower methylation (−6.6%) in a DMR in a putative bidirectional promoter and gene body in temporal and cerebral cortex (132) was replicated with lower methylation (−8%) in anterior cingulate gyrus and prefrontal cortex using the same platform (133). For *OR2L13*, the situation is different. Significantly higher promoter methylation (+18% at a DMP in PBLs) was seen in one study (136), whereas significantly lower methylation (−8% for the same probe within a DMR in buccal epithelium) was seen using a similar platform (130). Citing an additional study that found opposite directions of effect for ASD-associated expression of *OR2L13* (62), the latter study suggested that this gene may be especially epigenetically labile (130).

Two differentially methylated gene pathways were found in multiple studies: neurodevelopment and immune and inflammatory response (132, 134). The former were also found in genomic and transcriptomic studies of ASD and the latter mainly in transcriptomic and physiological studies. Of note, though, a recently compiled network of genes associated with a variety of genetic changes in ASD-affected individuals includes the complement genes *C1Q* and *C3* (184), which are also prefrontal cortex DMPs in the methylation study of Nardone and colleagues (133). Although there have been a greater number of epigenomic studies than transcriptomic studies of ASD, it is possible that immune-related pathways are more likely to be affected by the environment and/or stochastic processes influencing epigenetic state (132). Specific symptoms of immune dysfunction in ASD include neuroinflammation, presence of autoantibodies and enhanced T cell, natural killer cell and monocyte immune responses (51), and an increased frequency of allergies and autoimmune disorders together (185). Microglia are the major immune cell type in the brain and play major roles in neurodevelopment and synaptic function (186). Microglia may provide a mechanistic contribution to a proposed environmental cause of ASD, maternal immune activation (3). Maternal infection has also been involved in gene–environment interaction in ASD (187) and microglial activation has been observed in post-mortem brains from children with ASD (188). The *RORA* gene, found to be epigenetically different in ASD (134) also represents a gene with dual roles in immune function and neurodevelopment. Time will tell whether there are more pathways, such as oxidative phosphorylation and protein translation (131), that are shown to be consistently preferentially associated with epigenetic change in ASD and whether these are related to altered physiology in ASD.

To summarize, we present a possible model of the findings reviewed above. Figure 1 illustrates how genetic and epigenetic loads combine and interact to cross a threshold, over which, neurodevelopment, immune function and other pathways are compromised, neurodevelopmental buffering is ineffective and decanalization occurs. These dysfunctions are proposed to result in ASD, with individual phenotypes differing in a manner dependent on the specific gene networks compromised and in the timing and nature of each specific environment-induced set of epigenetic changes (i.e., unique ASD epigenomes).

## Future Directions

The five candidate genes whose methylation correlates with a diagnosis of ASD and for which there is additional evidence of involvement with ASD warrant attempts at replication, ideally in larger sample numbers and multiple tissues. Priority should be given to those with the largest effect sizes (*OXTR*, *EN2* and *MECP2*). In addition, all four DMPs/DMRs identified by two independent genome-wide studies also warrant such attempts at replication. This needs to be done before firm conclusions can be made about the association of these genes with causes and mechanisms of ASD (all tissues) and their suitability as ASD biomarkers (peripheral tissues). It is pertinent that all ASD DMRs identified in buccal epithelium (130) were also expressed in the brain, as were many of the ASD DMPs identified in blood (136).

Studies of genome-wide DNA methylation in ASD need to be larger, phenotyping needs to be standardized between studies, analysis platforms need to move toward true genomic coverage, genetic variation needs to be taken into account, and analytical methods need to be standardized to include differentially methylated probes and regions. In addition, with sufficient sample size, endophenotypes should be studied to reduce the genetic and epigenetic complexity. This will lead to systems-based approaches of integration of data from genomic, epigenomic, transcriptomic, proteomic and other platforms in more accurate statistical models (classifiers) of diagnosis, prognosis and risk estimation. This will also lead to a better understanding of gene–gene (29) and gene–environment interactions (69) in ASD. Clearly, autism researchers, and ultimately individuals with ASD, may have a lot to gain from the study of epigenetics, including the generation of robustly replicated epigenetic biomarkers for risk, diagnosis and prognosis of ASD, which may also be used to monitor response to future interventions (71).

## Conflict of Interest Statement

The authors declare that the research was conducted in the absence of any commercial or financial relationships that could be construed as a potential conflict of interest.
